# Comment on ‘Change in Physical Activity and Its Association With Decline in Kidney Function: A UK Biobank‐Based Cohort Study’ by Liu et al.

**DOI:** 10.1002/jcsm.13623

**Published:** 2024-10-21

**Authors:** Zhenzhi Qin, Yan Xu

**Affiliations:** ^1^ Department of Nephrology The Affiliated Hospital of Qingdao University Qingdao China; ^2^ Department of Pharmacology Qingdao University School of Pharmacy Qingdao China

We read with great interest the recent article by Welsh et al. titled ‘Change in physical activity and its association with decline in kidney function: A UK Biobank‐based cohort study’ in *Journal of Cachexia, Sarcopenia and Muscle* [1]. The study finds that increased physical activity may protect kidney function, as suggested by the modest yet significant associations observed in large‐scale analyses using eGFRCysC measurements. However, we note several biases in the use of the Cox proportional hazards (CoxPH) model that the authors did not address.

The established criteria may result in mixed censoring outcomes, that is, right‐censoring and interval‐censoring events [2, 3]. Events of kidney function diagnosed through medical records could result in interval‐censoring if they occurred between follow‐up visits, and right‐censoring for diagnosed between the end of follow‐up and the time of data analysis. The CoxPH model primarily handles right‐censored data. In contrast, the accelerated failure time (AFT) model is often preferred for scenarios involving various types of censored data [4]. The AFT model can effectively handle left‐censored, right‐censored and interval‐censored data by appropriately adjusting the likelihood function [5]. By using the ‘survival’ and ‘icenReg’ packages, mixed censored data can be fitted and analysed, and event times can be estimated [6].

Moreover, the CoxPH model requires the proportional hazards assumption, meaning that covariate effects are constant over time [7]. If this assumption is violated, the model may not provide unbiased estimates of the coefficients, and the predictions may not be reliable. The authors should utilize Schoenfeld residuals or alternative methods to evaluate the proportional hazards assumption for the association between covariates and the risk of kidney function. Schoenfeld residuals are calculated as the differences between the observed and expected values of covariates at each failure time [8]. If the residuals exhibit a systematic change over time, it suggests that the effect of the covariate may be time‐dependent. When the proportional hazards assumption does not hold, authors should use a stratified Cox model, a Cox model with time‐varying effects, or an AFT model instead of the standard CoxPH model [4, 9].

In conclusion, we believe that a re‐evaluation considering the potential impact of censoring events and the proportional hazards assumption is necessary. Further research is anticipated to provide more empirical data and clearer insights into this field.

## Conflicts of Interest

The authors declare no conflicts of interest.

## Data Availability

The datasets used and/or analysed during the current study are available from the corresponding author on reasonable request.
